# Co-Design and Development of the SmilesUp Text Messaging Intervention Using Behavioral Theory to Support Parents of Children With Early Childhood Caries: Mixed Methods Study

**DOI:** 10.2196/72107

**Published:** 2025-11-18

**Authors:** Rebecca Chen, Michelle J Irving, Carrie Tsai, Bradley Christian, Harleen Kumar, Angela Masoe, Neeta Prabhu, Woosung Sohn, Heiko Spallek, Clara K Chow

**Affiliations:** 1 Population Oral Health, School of Dentistry, Faculty of Medicine and Health, University of Sydney, 2 Chalmers Street, Surry Hills, New South Wales, 2010, Australia; 2 Westmead Applied Research Centre, Faculty of Medicine and Health, University of Sydney, Surry Hills, Sydney, Australia; 3 School of Dentistry, Faculty of Medicine and Health, University of Sydney, Sydney, Australia; 4 Paediatric Dentistry Department, Sydney Dental Hospital, Oral Health Services, Sydney Local Health District, Surry Hills, Australia; 5 Centre for Oral Health Strategy, NSW Health Ministry of Health, St. Leonards, Australia; 6 Paediatric Dentistry Department, Oral Health Services, Western Sydney Local Health District, Westmead, Australia

**Keywords:** mobile health, mHealth, dental caries, prevention, co-design

## Abstract

**Background:**

Early childhood caries (ECC) remains a common childhood condition that affects 600 million children worldwide. Providing parents with support for oral health behavior change can address ECC risk factors and complement preventive clinical care. Mobile health (mHealth) text message programs that are co-designed and evaluated by parents and health professionals using behavior theory have been shown to be effective in improving oral health outcomes.

**Objective:**

This study aimed to describe the co-design process, development, and content evaluation of a text message program designed to promote oral health behavior change among parents of children diagnosed with ECC using the Behavior Change Wheel (BCW) framework.

**Methods:**

The *SmilesUp* mHealth program was co-designed with parents in 2 stages using the BCW, a widely used theoretical framework to underpin mHealth programs, recommended by the World Health Organization. Through focus groups with parents in phase 1, the BCW was used to understand parental perspectives by identifying barriers and enablers and selecting target behaviors that could be feasibly delivered within a mHealth intervention. Barriers and enablers were mapped to the relevant theoretical domains and behavior change technique (BCT) of the BCW. Phase 2 evaluated content acceptability, measured by understandability, usefulness, and appropriateness of the program through questionnaires with parents and health professionals. Highly rated messages were finalized into an algorithm for the SMS text message program.

**Results:**

In phase 1, the overall target behavior was parental behavior change to support good oral health, including oral hygiene, reduced dietary sugar intake, and bedtime routines for their children. The 5 intervention functions focused on education, modeling, persuasion, environmental restructuring, and enablement, and 16 BCTs focused on addressing the motivational enablers and knowledge gap barriers identified by the parents. A total of 111 draft health messages were developed and mapped to the BCTs. In phase 2, a total of 2045 reviews of the 111 draft messages were completed by parents (14/31, 45.2%) and health professionals (17/31, 54.8%). Parents rated 77.4% (86/111) and health professionals rated 61.2% (68/111) of the messages as understandable, useful, and accepted. The messages that were considered understandable, useful, and appropriate by both groups were incorporated into the *SmilesUp* 12-week semipersonalized SMS message program.

**Conclusions:**

The *SmilesUp* mHealth program uses behavioral theory to address knowledge gaps in tooth brushing, diet, and bedtime routines identified by parents. It provides parents with convenient, bite-sized nudges of information to support oral health–promoting behaviors in the home context. Robust content development and evaluation are crucial initial steps before further investments are made to conduct a clinical trial to assess the effectiveness of the program.

## Introduction

Early childhood caries (ECC) remains a complex childhood disease that affects 600 million children worldwide [1]. In Australia, children with the most severe cases of ECC may require hospital admission, impacting approximately one in every 250 children [2]. Parents play a key role in preventing ECC by changing known modifiable oral health lifestyle behaviors [3]. Providing mothers, fathers, carers, and parents (from here on referred to as “parents”) with adequate and appropriate support for this behavior change remains key to sustainably improve oral health outcomes for their children across the life course [3]. ECC disease management approaches that focus on prevention and behavior change through motivational interviewing have been implemented over the past decade [4]. However, these strategies are time-intensive, costly, and have had limited population reach [5]. The widespread use of mobile phone technologies offers a valuable opportunity for modern health systems to increase the reach for prevention programs through prevention-focused digital health interventions [6]. These interventions can help drive sustainable oral health behavior change at scale [7,8]. While there are existing oral health mobile apps available, most focus only on tooth brushing and have been created for school-aged children or young adults [9,10]. These apps fail to comprehensively address the interrelated risk factors (oral hygiene, diet, and bedtime routines) specific to the prevention of ECC. Furthermore, many of these apps require the use of paid data, which can be prohibitive for some lower socioeconomic families, exacerbating potential disparities in digital health literacy [11,12]. Selecting an affordable medium to deliver digital health interventions can support equitable access to digital behavior change support among vulnerable populations [11]. Thoughtfully co-designed SMS interventions informed by behavioral theory have shown promise in improving general health behavioral outcomes, equitably and at scale [13-15]. To ensure practicability, mobile health (mHealth) programs should be compatible with any SMS text message systems used within existing digital health infrastructure. Making message delivery free or low cost will support equitable access to digital oral health interventions for diverse families at risk of ECC.

Involving and consulting consumers from the inception of the *SmilesUp* program development is crucial for developing effective content and features of the mHealth program [16]. Understanding the needs and preferences of parents enables health systems to develop mHealth interventions that genuinely support the health goals of the target population [17]. Furthermore, the latest World Health Organization (WHO) guidelines on mHealth emphasize the importance of a pragmatic approach, where developers of mHealth programs must balance consumer needs with the existing digital infrastructure and budget constraints within health systems [7]. Involving parents, health professionals, and policymakers early in the development process is crucial for creating feasible and scalable prevention-focused oral mHealth interventions [18].

Many co-designed mHealth programs supporting behavior change have used behavioral theory and content evaluation to ensure a robustly designed program [19-22]. The Behaviour Change Wheel (BCW) framework is a widely used theory for designing mHealth and digital health interventions [23-27]. It takes into account the broader social and legislative contexts, as well as societal limitations that may affect the desired outcomes of the target population [28]. Recently, the oral health academic community has recognized the importance of using the BCW to underpin the development and evaluation of behavior change interventions, including mHealth programs [29,30]. Similarly, the WHO guidelines emphasize the use of the BCW’s Capability, Opportunity, Motivation-Behaviour (COM-B) model as the comprehensive behavior change theory of choice for intervention development [28]. By using behavioral theory and content evaluation, interventions can comprehensively address barriers and enablers related to oral health behaviors identified by both parents and health professionals within health systems to reduce the ECC burden[7]. This mixed methods study aimed to use BCW behavioral theory to co-design and evaluate the content acceptability of *SmilesUp*, an SMS-based mHealth intervention for parents of children with ECC.

## Methods

### Participants and Recruitment Setting

The study was conducted in public dental services in New South Wales (NSW), targeting families receiving care and the oral health staff working with these families at 2 tertiary pediatric referral centers. NSW Health public dental services offer a range of safety-net dental services, including general anesthetic appointments for children who meet an eligibility criterion [31]. The service also prioritizes emergency situations and vulnerable populations at highest risk of dental disease [31,32].

Recruitment of parents for the co-designed workshops was conducted through recruitment flyers in the waiting rooms of the pediatric dentistry tertiary centers. Health professionals were also recruited via a flyer that was circulated via email from the heads of departments at the 2 tertiary centers and the state-based health promotion and policy team. Purposive sampling of parents with a child diagnosed with ECC accessing NSW public dental services ensured representation of the end-user perspectives of the final *SmilesUp* program. Similarly, only health professionals who were working within NSW public dental clinics or for the state-wide oral health promotion team were invited to participate.

### Ethical Considerations

Ethics approval was obtained and approved by the Western Sydney Local Health District Research ethics department (2022/ETH01920). As part of the recruitment process, all participants for both phases were given a participant information sheet and provided e-consent through Research Electronic Data Capture (REDCap) or via a paper consent form before joining workshops, semistructured interviews, or completing the questionnaire. For participants attending in person focus groups, recurtiment flyers outlined that they would be reimbursed for their travel and parking expenses; no other compensation was provided. All research data collected were deidentified and procedures were conducted in alignment with the National Statement on Ethical Conduct in Human Research from the Australian National Health and Medical Research and the Declaration of Helsinki.

### Overview of the Co-Design Process

**Figure 1.**
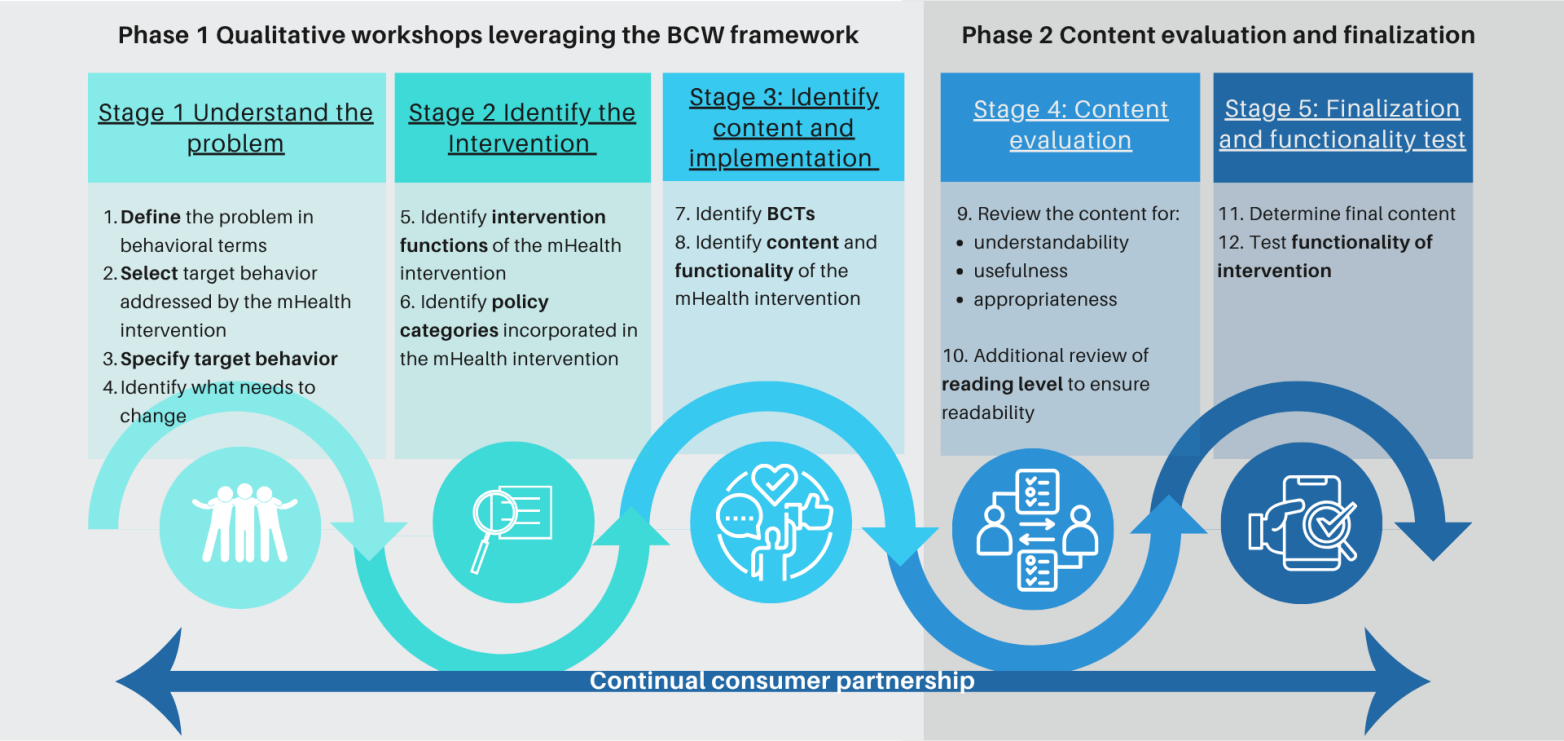
SmilesUp mobile health (mHealth) intervention co-design process, including the Behaviour Change Wheel (BCW) framework and content evaluation and finalization. BCT: behavior change technique.

This study used a 2-phase co-design approach, incorporating BCW and a mixed methods evaluation similar to previously published methodologies [21,33]. Phase 1 involved qualitative methods [34] to understand the oral health barriers and enablers faced by parents, identify target behaviors, and draft intervention content. Phase 2 focused on evaluating content validity by measuring understandability, usefulness, and appropriateness with both parents and health professionals (Figure 1).

### Phase 1: Qualitative Workshops Leveraging BCW

#### Overview of Phase 1

Phase 1 used all 3 stages of BCW (Figure 1) to understand parents’ barriers and enablers, mapping these to intervention functions, behavior change techniques (BCTs), and text message content. Co-design workshops and semistructured interviews were conducted between February 2, 2023, and July 15, 2023. Data were collected by a trained qualitative researcher who had experience working within NSW Health public dental services, and sessions were conducted in person or via video calls if requested by participants. All workshop participants’ anonymity was maintained through pseudonyms, and transcription was managed with the NVivo (Lumivero) qualitative data analysis software [35]. Parent and health professional sessions were held separately to reduce power dynamics and encourage open discussions [36,37].

#### Stage 1: Understanding the Problem

In the first stage, qualitative workshops and interviews were conducted with parents and oral health professionals to explore barriers and enablers related to 3 key oral health behaviors: oral hygiene, dietary sugar intake, and bedtime routines. These were mapped to the COM-B model to define a target behavior that could be supported by mHealth messaging.

#### Stage 2: Identifying the Intervention Options and Policy Areas

Stage 2 used the BCW mapping process to select intervention functions and policy areas to address the barriers and enablers identified in stage 1. These identified intervention functions also had to have the capacity to be incorporated into the format of the mHealth program.

#### Stage 3: Identifying BCTs and Drafting Content

In stage 3, BCTs were chosen based on the Acceptability, Practicability, Effectiveness, Affordability, Safety, and Equity (APEASE) [38] criteria, and content for the intervention was drafted and mapped to the original barriers and enablers. Within the BCW, the APEASE criteria ensures that the content is pragmatic and relevant for both the parents and the health system’s existing digital infrastructure [28]. This process informed the content of the initial draft text message bank.

### Phase 2: Content Evaluation and Final Functionality

#### Overview of Phase 2

Phase 2 developed the information gathered from the phase 1 co-design workshops by conducting a broader consultation with parents and health professionals to further evaluate the content (stage 4) before finalising the content and functionality of the *SmilesUp* program (stage 5).

#### Stage 4: Content Evaluation by a Broader Range of Parents and Health Professionals

A questionnaire based on previously published methodologies [15] was developed to measure the understandability, usefulness, and appropriateness of the draft content. This questionnaire was given to a wider group of parents and health professionals to assess the acceptability of SMS text message content [15]. We recruited until all messages were reviewed by at least 2 parents and 2 health professionals. The SMS text message content reviews were collected through questionnaires on REDCap and through paper forms when requested by parents [39]. To prevent survey fatigue, each participant was only required to provide feedback on the understandability, usefulness, and appropriateness of approximately 20 messages [15]. Additionally, to ensure the readability of the messages, a grade 5 reading level or below was set to ensure the readability of the program for our target population. All final messages were assessed using the Sydney Health Literacy Lab (SHeLL) reader to verify this reading level was met before they were included in the final set [40].

#### Stage 5: Finalization and Functionality Determination

In the finalization phase, messages were mapped back to the originally identified barriers and enablers (phase 1) to ensure that all identified needs and BCTs were addressed. Qualitative data were also analyzed to inform decisions about the program’s functionality, including the duration, frequency, timing, and sequencing of the SMS text message schedule.

## Results

### Participants

Members included in the co-designed workshops were parents 30% (6/20) currently using public dental services, as well as health professionals 70% (14/20), including dental practitioners, health promotion policy officers, dietitians, and IT managers providing clinical services or developing programs for oral health services within NSW Health. This diverse group provided a broad perspective, shared existing high-quality digital health promotion resources (interactive websites or online videos) that could be incorporated into the program, and guided the features of the program to address target behaviors. The inclusion of various dental practitioners and dietitians ensured a comprehensive approach to oral health promotion information that addressed the specific barriers and enablers identified by parents. Additional parents (14/31, 45.2%) and health professionals (17/31, 54.8 %) were recruited for phase 2 to ensure a broader perspective beyond that of the initial co-design groups.

### Phase 1: Co-Design Outcomes Using BCW

The results from the mixed methods data collection and analysis were progressive, integrated, and structured according to our methods (Figure 1). Phase 1 results are presented within the 3 stages set by BCW [28].

#### Stage 1: Understanding the Behavior

Workshops with parents and health professionals identified 15 enablers and 10 barriers to address 3 key oral health risk behavioral categories: oral hygiene, diet, and healthy bedtime routines. These barriers and enablers were mapped to BCW’s COM-B model of behavior categories: *physical capability*, *psychological capability*, *physical opportunity*, *social opportunity*, *reflective motivation*, and *automatic motivation*. We found that while parents had the *physical capability* to perform oral health–promoting behaviors, they lacked specific knowledge (*psychological capability*) about selecting the best toothpaste, brushing techniques, and choosing low sugar snacks for their children. Most of the enablers were linked to *motivation* (*automatic*) and *capability* (*knowledge*) where parents sought reminders and information to help them develop consistent oral health habits.

Therefore, the problem was defined as the need to increase parents’ oral health literacy encompassing both knowledge (*psychological capability*) and motivation (*automatic and reflective*) for adopting healthy oral behaviors to prevent ECC. A comprehensive tabulated description of the target behavior for the *SmilesUp* SMS text message program using BCW framework is found in Table 1.

**Table 1.** Using the Behavior Change Wheel (BCW) framework to identify the specific target behavior for the *SmilesUp* SMS text message program.

Question (BCW step)	Response
What is the target behavior?	Participation in a structured SMS messaging intervention to perform oral health–promoting behaviors in the home context.
Who needs to perform the behavior?	Parents of children with ECC^a^, undergoing dental treatment within select NSW^b^ Health public dental clinics, including but not limited to children waiting for a general anesthetic appointment for dental treatment.
What does the person need to do differently to achieve the desired change?	Participate in a co-designed and semipersonalized structured SMS messaging program that supports a range of healthy oral health behaviors, including oral hygiene, diet, and bedtime routines.
When will they do it?	Parents will engage with the SmilesUp 12-week program after their initial clinical appointment, which provides additional support to the oral hygiene and dietary advice provided by their clinicians, with messages sent at times nominated by the parents at the start of the program.
Where will they do it?	In the home context.
How often will they do it?	The program will be sent 2‐3 times per week. However, most key behaviors related to ECC (eg, tooth brushing) will need to be conducted daily.
With whom will they do it?	With their children and other relevant members of their family.

a ECC: early childhood caries.

b NSW: New South Wales.

#### Stage 2: Intervention Options and Policy Areas Identified

Intervention options and policy areas from the BCW were identified based on the initial data around barriers and enablers collected from parents and clinicians. These were then mapped to the previously identified barriers and enablers (MultimediaAppendices 1  2). Parents and clinicians recommended incorporating 5 intervention functions and 10 theoretical domains into the *SmilesUp* mHealth intervention. The 5 intervention functions were education, persuasion, modeling, enablement, and environmental restructuring [38]. The 10 theoretical domains framework included knowledge, skills, beliefs about capabilities, optimism, beliefs about consequences, reinforcement, intentions, goals, memory, and social influences [38].

The *SmilesUp* program aligns with two specific policy areas identified in the BCW: (1) communication and marketing and (2) service provision [38]. The program sits at the intersection of these policies because it is delivered as a communication tool to support and encourage behavior change as part of the comprehensive, value-based clinicalservice provided to families with the NSW public dental service.

#### Stage 3: BCTs and Drafting Content Identified

Specific BCTs were determined using a feasibility lens based on the APEASE criteria with perspectives from parents and high-level stakeholders [28]. A total of 16 BCTs were identified and mapped to the 15 enablers and 10 barriers previously determined by the consumer co-designers in stages 1 and 2 (MultimediaAppendices 1  2). These BCTs were chosen for their feasibility and relevance to an mHealth intervention with the goal of enhancing parents’ capability and motivation of oral health–promoting behaviors in the home context (MultimediaAppendices 1  2). The specific BCTs included were as follows: “1.4 Action planning”; “2.4 Self-monitoring of behavior”; “4.1 Instructions on how to perform behavior”; “5.1 Information about health consequences”; “5.3 Information about social and environmental consequences”; “5.6 Information about emotional consequences”; “6.1 Demonstration of the behavior”; “7.3 Reducing the prompts/cues”; “7.6 Satiation”; “8.2 Behavioral substitution”; “8.3 Habit formation”; “9.1 Credible source”; “12.1 Restructuring the physical environment”; “12.4 Distraction”; “13.1 Identification of self as a role model”; “15.1 Verbal persuasion about capability.” An example of how the BCTs were comprehensively mapped to the barriers or enablers, theoretical domains framework, and policy functions is provided in MultimediaAppendices 1  2. This information informed the program’s functionality, including the need for semipersonalization of the messages to include the child’s name, and for parents to select the timing of the messages to suit their lifestyles, thereby supporting ongoing engagement and sustained long-term behavior change.

The draft content, co-designed with 20 participants including 6 (30%) parents and 14 (70%) health professionals, addressed the barriers and enablers and corresponded to the intervention functions and BCTs identified earlier. The draft message bank included 111 messages, some of which were adapted from the bank of messages from the WHO’s *BeHealthy BeMobile* program, with locally relevant web-based links specific to the target population [7]. These web-based resources were in alignment with Australia’s National Consensus Statement on Oral Health Messages [41] and were developed by government agencies and professional associations including NSW Health and the Australian Dental Association [41]. New messages were created to address specific knowledge barriers identified by the parents, such as the consumption of dried fruit and vitamin gummies that had been noted in stage 1. Additionally, BCTs were incorporated into the content and features of the mHealth program to support parents using the final *SmilesUp* program.

### Phase 2: Content Evaluation and Final Functionality Determination

#### Stage 4: Content Evaluation Findings by a Broader Range of Parents and Health Professionals

In phase 2, of the 31 participants, 14 (45.2%) parents and 17 (54.8%) health professionals reviewed the 111 draft SMS text messages, providing a total of 2045 ratings, which included 805 (39.4%) parent reviews and 1240 (60.6%) health professional reviews. The sample size was determined by data saturation, which was reached when all messages were reviewed twice—once by a health professional and once by a parent consumer accessing NSW public dental clinics. For a message to be considered acceptable, it needed to receive a “yes” favorably on all 3 categories of understandability, usefulness, and appropriateness. Of the 111 draft messages, 86 (77.5%) messages were rated favorably by parents, while 68 (61.3%) messages were rated favorably by health professionals. A total of 56 (50.5%) messages were considered acceptable and useful by both parents and health professionals.

These 56 messages were further tested for readability using the SHeLL reader [40]. Two (1.8%) messages that exceeded the grade 5 reading level were removed.

#### Stage 5: Program Finalization and Functionality Determination

The final set of messages was mapped to oral health behaviors, barriers, enablers, intervention functions, and BCTs. Based on the final functionality determination, only 36 messages were required. Therefore, 18/56 (32%) messages that addressed similar barriers and enablers were removed to avoid redundancy. The final set included 12/36 (33.3%) messages addressing oral hygiene, 16/36 (44.4%) addressing dietary sugar intake, and 6/36 (16.7%) on bedtime routines. An example of how a barrier is mapped to an intervention function, a BCT and the SMS text message content for the program, is found in Table 2.

**Table 2.** Example of a barrier and enablers mapped to the Behaviour Change Wheel (BCW) framework and SMS text message content.

Barrier or enabler identified	COM-B^a^	TDF^b^ and intervention function	Policy function	BCT^c^	Text message examples
Forgetfulness (barrier)	Motivation (automatic)	Reinforcement and enablement	Communication or marketing service provision	2.3 Self-monitoring of behavior; 4.1 Instructions on how to perform behavior; 13.1 Identification of self as a role model; 15.1 Verbal persuasion about capability	“Have you watched your children brushing their teeth today, did they do a good job? Brushing your teeth together can help them learn great habits @SMILESup”
Receiving reminders and tips (enabler)	Motivation (automatic)	Reinforcement and enablement	Communication or marketing service provision	8.3 Habit formation	“A little reminder for you to help [child name] brush their teeth before they go to bed. If they have clean teeth before they sleep, it will prevent tooth decay. @SMILESup”

a COM-B: Capability, Opportunity, Motivation-Behaviour.

b TDF: Theoretical Domains Framework.

c BCT: behavior change technique.

Finalizing the program’s functionality included decisions about the duration, frequency, timing, and sequencing of the SMS text message algorithm. After consulting parents and considering the behavior change literature [42], the program was set for 12 weeks with an average of 3 messages per week, totaling 36 messages. Parents could semipersonalize the program by including their child’s name in the messages and choosing the time they received the messages to best fit their lifestyles. The sequencing initially focused on bridging knowledge gaps identified by parents during the first month, with more reminders and messages targeting motivation scheduled for later in the program.

The message schedule and practice sequence were delivered using the Westmead Applied Research Centre’s TextCare platform, a cloud-based system designed to semipersonalize the content and frequency of SMS text messages. TextCare includes built-in safety, privacy, and security systems with in-built data analytics, including an automated opt-out feature to measure engagement from parents. The messages are primarily 1-way, providing practical tips and strategies to prompt behavior change. The TextCare platform was monitored by a clinician researcher. RC tested the program for 1 month before delivering the program to parents.

## Discussion

### Principal Findings

This study reports the outcomes of a co-design and content evaluation of the *SmilesUp* SMS text message intervention, aimed at supporting parents of children with ECC in promoting oral health behavior change. Guided by the BCW, the intervention incorporates 16 BCTs to address motivation and knowledge gaps identified by parents. Of the 111 drafted messages, 56 (50.5%) text messages that were evaluated to be understandable, useful, and acceptable by both parents and health professionals were programmed into the 12-week semipersonalized SMS text message program. This paper highlights the importance of involving parent end users at the beginning of the design process to ensure that the intervention addresses their needs in developing oral health–promoting behaviors for their children. This co-design work lays the foundation for future studies to test the effectiveness of the mHealth intervention for changing oral health behaviors [43].

### Strengths and Limitations

A major strength of our study is the robust co-design process, structured around the BCW behavioral theory using mixed methods to incorporate perspectives from both parents and health professionals. The BCW, including the COM-B model of behavior change, ensured that the target behaviors were matched with the 16 BCTs to address identified barriers and enablers. The study aligns with the WHO’s goal of optimizing digital technologies for oral health within its Draft Global Oral Health Action Plan (2023‐2030) and serves as an example of how a mHealth program can address ECC localized to the Australian context [44].

Methodologically, our study builds on the existing methods from other lifestyle-related behavioral studies [15,21,25,45-47] and is one of the few studies in the field of dentistry using a theory-driven design for mHealth programs focused on behavior change [48-50]. Our process was pragmatic, systematic, and grounded in behavioral theory. Although the formal triangulation of data was not conducted, qualitative findings informed content development, which subsequently guided the quantitative data collection. The quantitative insights were subsequently synthesized with the initial qualitative findings to ensure alignment with the original aims of the program. This iterative process led to the development of a distinctive program that addressed multiple oral health risk factors. Previous mHealth programs in oral health tended to focus on single risk behaviors, for example, on bedtime routines [49] or on diet [51]. Although specific to the Australian context, the robustness and adaptability of this program enables other countries to also co-design, adapt, and localize an mHealth program. This is especially important in lower resourced areas, including low- and middle-income countries in the Asia-Pacific region [52] and more globally [7,53]. Where both oral health consumers and practitioners are increasingly using digital technologies to enhance the preventive potential of oral health[54]. Furthermore, by using the BCW, this study contributes to the dental scientific community’s understanding of effective BCT approaches used to improve oral health–related behaviors in the long term [20,29,30].

Raising the standard of mHealth design and involving end users in the design process is essential to ensure confidence in the impact that mHealth interventions can have on improving health outcomes. Continual consultation with parents shaped the content and functionality of the *SmilesUp* program, ensuring it was patient centered and designed for vulnerable families using NSW Health public dental clinics [18]. This approach aligns with value-based health care systems that prioritize the consumer voice [55] and enhances confidence in mHealth interventions and their potential impact. By using co-design and content evaluation, the *SmilesUp* program completed content acceptability testing to inform policy and funding decisions before further investments are made for efficacy testing.

A limitation of the current *SmilesUp* program is the 1-way SMS text messaging format. Although newer mobile health tools like mobile apps and wearables seem more advanced [56], our use of the APEASE framework and consumer consultation shows that SMS text messaging still remains an effective, equitable, and widely accepted option for patients and practical for health systems [15,57-60]. Furthermore, compared with standalone mobile apps, the TextCare platform enables analytics on message delivery and replies to assess the reach and engagement of the program. These data are critical for a process and feasibility evaluation before health systems make larger investments into digital health infrastructure to further embed programs like *SmilesUp* into their existing digital health management systems [56,61].

### Future Directions

This co-design work is the first step before we conduct future research to test the effectiveness of the program in improving oral health behavioral outcomes, especially among parents of children at a high risk of ECC [43,62,63]. The effectiveness trial will be conducted in public dental care settings using the COM-B model to inform analytical strategies and outcome measures [43,64,65]. Future studies will also examine opportunities to expand the program for broader reach by translating the content to other languages and scaling the program to reach more families.

### Conclusions

*SmilesUp* is a robust, co-designed, and behavioral theory–driven mHealth intervention developed to support parents, especially those from vulnerable and low socioeconomic families, to adopt key oral health behaviors to prevent ECC. By involving end-user parents in the co-design process, applying behavioral theory, and evaluating the content, the program ensures that it addresses specific knowledge gaps related to brushing, diet, and bedtime routines. This is a crucial first step before conducting a planned future trial to test the effectiveness of the mHealth intervention for behavior change and the prevention of ECC.

## Supplementary material

10.2196/72107 Multimedia Appendix 1 Barriers identified by parents and health professionals mapped to the Behaviour Change Wheel framework.

10.2196/72107 Multimedia Appendix 2 Enablers identified by parents and health professionals mapped to the Behaviour Change Wheel framework.

## Abbreviations

APEASE: Acceptability, Practicability, Effectiveness, Affordability, and Equity
BCT: behavior change technique
BCW: Behaviour Change Wheel
COM-B: Capability, Opportunity, Motivation-Behaviour
ECC: early childhood caries
mHealth: mobile health
REDCap: Research Electronic Data Capture
SHeLL: Sydney Health Literacy Lab
WHO: World Health Organization
