# Supplementary material for: Co-Design and Development of the SmilesUp Text Messaging Intervention Using Behavioral Theory to Support Parents of Children With Early Childhood Caries: Mixed Methods Study
Source: JMIR Pediatr Parent. 2025 Nov 18;8:e72107. doi: 10.2196/72107 (PMC12626244; doi:10.2196/72107)
Supplement: Multimedia Appendix 1 [file pediatrics-v8-e72107-s001.docx]

| **Multimedia Appendix 1: Barriers identified by parent and health professionals mapped to the BCW (Behaviour Change Wheel) framework** | | | | |
| --- | --- | --- | --- | --- |
| **Barrier selected** | **COM-B model of behaviour category** | **TDF Domain:** | **Relevant intervention function** | **BCT used within the SMS intervention**  **This has been labelled from the BCTT taxonomy. Behaviour Change Technique Taxonomy attached to each message (BCTT)** |
| **Oral Hygiene** | | | | |
| 1.Lack of knowledge related oral hygiene practices |  |  |  |  |
| 1.1Lack of knowledge (about toothpastes, including which to purchase, and mint alternative flavoured toothpastes) | Capability psychological | Knowledge | Education | 4.1 Instructions on how to perform behaviour 9.1 Credible source |
| 1.2Lack of knowledge (about tooth brushing technique) | Capability psychological | Skill | Training | 6.1 Demonstration of the behaviour |
| 1.3Lack of knowledge (about how much toothpaste to put on a brush) | Capability psychological | Knowledge | Education | 4.1 Instructions on how to perform behaviour 6.1 Demonstration of the behaviour |
| 1.4 Lack of knowledge (about what type of toothbrush) | Capability psychological | Knowledge | Education | 4.1 Instructions on how to perform behaviour |
| 1.5Lack of knowledge (about when to brush? In the morning before or after breakfast?) | Capability psychological | Knowledge | Education | 4.1 Instructions on how to perform behaviour |
| 1.6Lack of knowledge (about how long toothbrushing should be?) | Capability psychological | Knowledge | Education | 4.1 Instructions on how to perform behaviour |
| 1.7Lack of knowledge about long term oral health consequences | Capability psychological | Beliefs about consequences | Education/ persuasion | 5.1 Information about health consequences |
| 2. Lack of motivation | Motivation reflective | Beliefs about capabilities | Persuasion / Enablement | 5.6 Information about emotional consequences  13.1 Identification of self as role model 15.1 Verbal persuasion about capability |
| 3. Forgetfulness | Motivation automatic | Reinforcement | Enablement | 2.3 Self-monitoring of behaviour  13.1 Identification of self as a role model |
| **Food and drink** | | | | |
| 4. Beliefs about capabilities  4.1 Fatalistic beliefs of parents who believe that they can't change oral health outcomes for their child but can change other health outcomes. | Motivation reflective | Beliefs about capabilities | Persuasion / Enablement | 4.1 Instructions on how to perform behaviour 15.1 Verbal persuasion about capability |
| 4.2 Parents see oral health is not important as other priorities | Motivation reflective | Knowledge | Education/ Persuasion | 5.3 Information about social and environmental consequences |
| 5. Lack of knowledge about healthy foods and drinks |  |  |  |  |
| 5.1 Lack of knowledge (about healthy snack/food options, especially when purchasing snacks at the shops) | Capability psychological | Knowledge | Education | 4.1 Instructions on how to perform behaviour  6.1 Demonstration of the behaviour  8.2 Behavioural substitution |
| 5.2 Lack of knowledge (about muesli bars and other sticky snack foods) | Capability psychological | Knowledge | Education | 5.1 Information about health consequences  8.2 Behavioural substitution |
| 5.3 Lack of knowledge (about reading food labels and understanding the amount of sugar in foods) | Capability psychological | Knowledge | Education | 4.1 Instructions on how to perform behaviour |
| 5.4 Lack of knowledge (about the problems associated with vitamin gummies and alternative options for providing children with adequate vitamin intake) | Capability psychological | Knowledge | Education | 5.1 Information about health consequences  8.2 Behavioural substitution |
| 5.5 Lack of knowledge (about fruit juices and healthier alternatives) | Capability psychological | Knowledge | Education | 5.1 Information about health consequences  8.2 Behavioural substitution |
| 5.6 Lack of knowledge (about the oral health implications of consuming too much dried fruit) | Capability psychological | Knowledge | Education | 5.1 Information about health consequences  8.2 Behavioural substitution |
| 6. Parents feeling the peer pressure of children comparing their lunch with what other children have in school lunches | Opportunity social | Social influences | Education/ Persuasion | 6.2 Social comparison (to healthy role models) |
| 7. Children pressuring parents to purchase certain snacks whilst shopping | Motivation reflective | Beliefs about capabilities | Persuasion / Enablement | 1.4 Action Planning  4.1 Instructions on how to perform behaviour  8.2 Behavioural substitution  12.1 Restructuring the physical environment |
| **Bedtime routine** | | | | |
| 8. Lack of knowledge about the importance of bedtime routines and oral health | Motivation reflective | Beliefs about capabilities | Persuasion / Enablement | 4.1 Instructions on how to perform behaviour 5.1 Information about health consequences  8.3 Habit formation  15.1 Verbal persuasion about capability |
| 9. Interferes with other non-oral health promoting bedtime routines | Motivation reflective | Intentions | Persuasion / Enablement | 4.1 Instructions on how to perform behaviour  5.1 Information about health consequences |
| 10. Parents acknowledge that habit formation is difficult and requires reminders. | Motivation automatic | Goals | Persuasion / Enablement | 2.4 Self-monitoring of behaviour  5.1 Information about health consequences  8.3 Habit formation  12.4 Distraction – e.g. songs to distract children whilst performing oral hygiene behaviours. |
