# Supplementary material for: Co-Design and Development of the SmilesUp Text Messaging Intervention Using Behavioral Theory to Support Parents of Children With Early Childhood Caries: Mixed Methods Study
Source: JMIR Pediatr Parent. 2025 Nov 18;8:e72107. doi: 10.2196/72107 (PMC12626244; doi:10.2196/72107)
Supplement: Multimedia Appendix 2 [file pediatrics-v8-e72107-s002.docx]

| **Multimedia Appendix 2: Enablers identified by parents and health professionals mapped to the BCW (Behaviour Change Wheel) framework** | | | | |
| --- | --- | --- | --- | --- |
| **Enabler selected**  **Enabler with specific content** | **COM-B model of behaviour category** | **TDF Domain** | **Relevant intervention function** | **BCT used within the SMS intervention** |
| **Oral hygiene** | | | | |
| 1.Brushing together/supervised brushing | Opportunity social | Knowledge/social influences | Modelling | 2.4 Self-monitoring of behaviour 4.1 Instructions on how to perform behaviour 13.1 Identification of self as role model 15.1 Verbal persuasion about capability |
| 2. Making oral hygiene routines fun | Motivation automatic | Reinforcement | Persuasion | 12.4 Distraction – e.g. songs to distract children whilst performing oral hygiene behaviours. |
| 3. Prioritising oral health behaviours | Motivation reflective | Intentions  Reinforcement  Knowledge | Education/ persuasion | 1.4 Action Planning |
| 4. Reassurance - belief that you can change oral health outcomes for your child | Motivation reflective | Reinforcement/Knowledge/  Memory | Education/ persuasion | 5.6 Information about emotional consequences |
| 5. Receiving reminders and tips | Motivation automatic | Reinforcement | Enablement | 8.3 Habit formation |
| 6. Having all the correct equipment to perform tooth brushing | Opportunity physical | Environmental context | Environmental restructuring | 12.1 Restructuring the physical environment |
| 7. Having 'role models' performing healthy behaviours | Opportunity social | Social influences | Modelling | 6.2 Social comparison (to healthy role models) |
| 8. Believing that they (the parent) can be role model for their children | Opportunity social | Social influences | Modelling | 13.1 Identification of self as a role model  15.1 Verbal persuasion about capability |
| 9. Personalisation of messages | Motivation automatic | Knowledge / reinforcement | Enablement | 2.4 Self-monitoring of behaviour  15.1 Verbal persuasion about capability |
| **Food and drink** | | | | |
| 10. Linking oral health to other health consequences | Capability psychological | Knowledge | Education/ persuasion | 5.1 Information about health consequences |
| 11. Giving children choice about healthy food options | Motivational reflective | Beliefs about consequences | Education/ persuasion | 1.4 Action Planning 8.2 Behavioural substitution |
| **Bedtime routine** |  |  |  |  |
| 12. Providing nutritious filling meals at dinner time to reduce snacking | Capability psychological | knowledge | Education/ persuasion | 7.6 Satiation  8.2 Behavioural substitution |
| 13. Integrating brushing in the bedtime routine | Capability psychological | knowledge / reinforcement | Education/ persuasion | 4.1 Instructions on how to perform behaviour  12.4 Distraction |
| 14. Choosing the timing of the messages | Motivation reflective | reinforcement | Enablement | 8.3 Habit formation |
| 15. Enabling confidence by reducing the number of prompts and cues provided towards the end of the program | Motivation reflective | reinforcement | Enablement | 7.3 Reducing the prompts/cues |
